# Functional anatomy of a giant toothless mandible from a bird-like dinosaur: *Gigantoraptor* and the evolution of the oviraptorosaurian jaw

**DOI:** 10.1038/s41598-017-15709-7

**Published:** 2017-11-24

**Authors:** Waisum Ma, Junyou Wang, Michael Pittman, Qingwei Tan, Lin Tan, Bin Guo, Xing Xu

**Affiliations:** 1 Vertebrate Palaeontology Laboratory, Department of Earth Sciences, University of Hong Kong, Pokfulam, Hong Kong China; 2 Longhao Institute of Geology and Paleontology, Hohhot, Nei Mongol, 010011 China; 3 Nei Mongol Museum of Nature, Hohhot, Nei Mongol, 010011 China; 4 Key Laboratory of Vertebrate Evolution and Human Origins of Chinese Academy of Sciences, Institute of Vertebrate Paleontology and Paleoanthropology, Chinese Academy of Sciences, Beijing, 100044 China

## Abstract

The Oviraptorosauria are a group of theropod dinosaurs that diverged from the typical carnivorous theropod diet. It includes two main lineages – Caenagnathidae and Oviraptoridae – that display a number of differences in mandibular morphology, but little is known about their functional consequences, hampering our understanding of oviraptorosaurian dietary evolution. This study presents the first in-depth description of the giant toothless mandible of *Gigantoraptor*, the only well-preserved stemward caenagnathid mandible. This mandible shows the greatest relative beak depth among caenagnathids, which is an adaptation seen in some modern birds for processing harder seeds. The presence of a lingual triturating shelf in caenagnathids more crownward than *Gigantoraptor* suggests a possible increased specialization towards shearing along this lineage. Like other oviraptorosaurs, the possession of a dorsally convex articular glenoid in *Gigantoraptor* indicates that propalinal jaw movement was probably an important mechanism for food processing, as in *Sphenodon* and dicynodonts. Oviraptorid mandibles were more suited for producing powerful bites (e.g. crushing-related) compared to caenagnathids: oviraptorids generally possess a deeper, more downturned beak, a taller coronoid process prominence and a larger medial mandibular fossa. This disparity in caenagnathid and oviraptorid mandible morphology potentially suggests specialization towards two different feeding styles – shearing and crushing-related mechanisms respectively.

## Introduction

The Oviraptorosauria is a group of coelurosaurian theropod dinosaurs that are first recorded in the Aptian stage of the Early Cretaceous (~125 million years ago)^1,2^ (but some phylogenetic analyses suggest an earlier Middle-Late Jurassic age instead^3^) and became extinct at the end of the Cretaceous (~66 million years ago)^4^. They are often described as ‘bird-like’ as they possess several characteristics that are also found in living birds, most notably a beak^4^, but early oviraptorosaurs such as *Incisivosaurus gauthieri* ^1^, *Caudipteryx zoui* ^2^ and *Protarchaeopteryx robusta* ^2^ had teeth. Oviraptorosaurians are one of several theropod groups that appear to diverge from the ancestral carnivorous diet of theropods^5^. Earlier oviraptorosaur studies had variable opinions about diet, including suggestions of carnivory^6^, durophagy^7^ and herbivory^8^. Recent studies and discoveries tend to support the idea that at least some oviraptorosaurs were herbivorous^2,9–11^. Zanno & Makovicky^5^ inferred herbivory for the whole oviraptorosaurian clade based on a number of skeletal features related to herbivory. However, little is known about how feeding behaviour might have changed within the clade along its two main lineages – Caenagnathidae and Oviraptoridae^12,13^. Caenagnathids are known from both North America and Asia whereas oviraptorids have only been discovered in Asia^12^. Both of them possess bizarre cranio-mandibular features that deviate from typical theropods (e.g. toothlessness, skull pneumatisation and possession of a cranial crest and a relatively deep skull). However, the cranial differences between caenagnathids and oviraptorids are poorly understood, due to the rarity and fragmentary nature of caenagnathid skull material^14,15^. It has been suggested that oviraptorids preferred arid or semi-arid habitats, whereas caenagnathids preferred wetter, fluvial habitats^16^. These different environmental associations may indicate differences in their ecology^10,16^, or they could potentially be related, at least in part, to preservational artifacts.

This study presents a detailed description of the mandibular anatomy of the gigantic oviraptorosaur *Gigantoraptor erlianensis*. *Gigantoraptor* was initially placed at the base of the oviraptorid lineage^17^ and was later identified as a stemward caenagnathid^10^, as in later phylogenetic analyses^12,15,18,19^. *Gigantoraptor* is the only stemward caenagnathid with a well-preserved mandible, and the goal of this study is to use it to reconstruct the evolution of oviraptorosaur mandibular anatomy and function.

*Gigantoraptor* was recovered from the Upper Cretaceous Erlian Formation of Inner Mongolia, China in 2007^17^ (we use the most recent Chinese nomenclature for the Iren Dabasu Formation^20,21^). It is known from a single associated fragmentary skeleton consisting of a nearly complete mandible and some postcranial bones^17^. *Gigantoraptor* is estimated to have been much heavier than typical oviraptorosaurs: compared to the similar-aged Mongolian oviraptorid *Citipati osmolskae* it was ~20 times heavier (~2000kg compared to ~100 kg)^22^. It is estimated to have been even larger than Erlian’s tyrannosaur *Alectrosaurus* which only weighed ~600 kg^22^. Given its gigantic body size, *Gigantoraptor* also provides an opportunity to determine how size may have been related to its feeding strategy. In deepening our understanding of oviraptorosaur mandibular anatomy and function and possible size-related factors affecting it, further insights can be gained into how dietary shifts occurred within Theropoda, potentially clarifying aspects of the complex range of convergent and unique evolutionary changes that appear to have occurred^5,23^.

## Description

**Figure 1:**
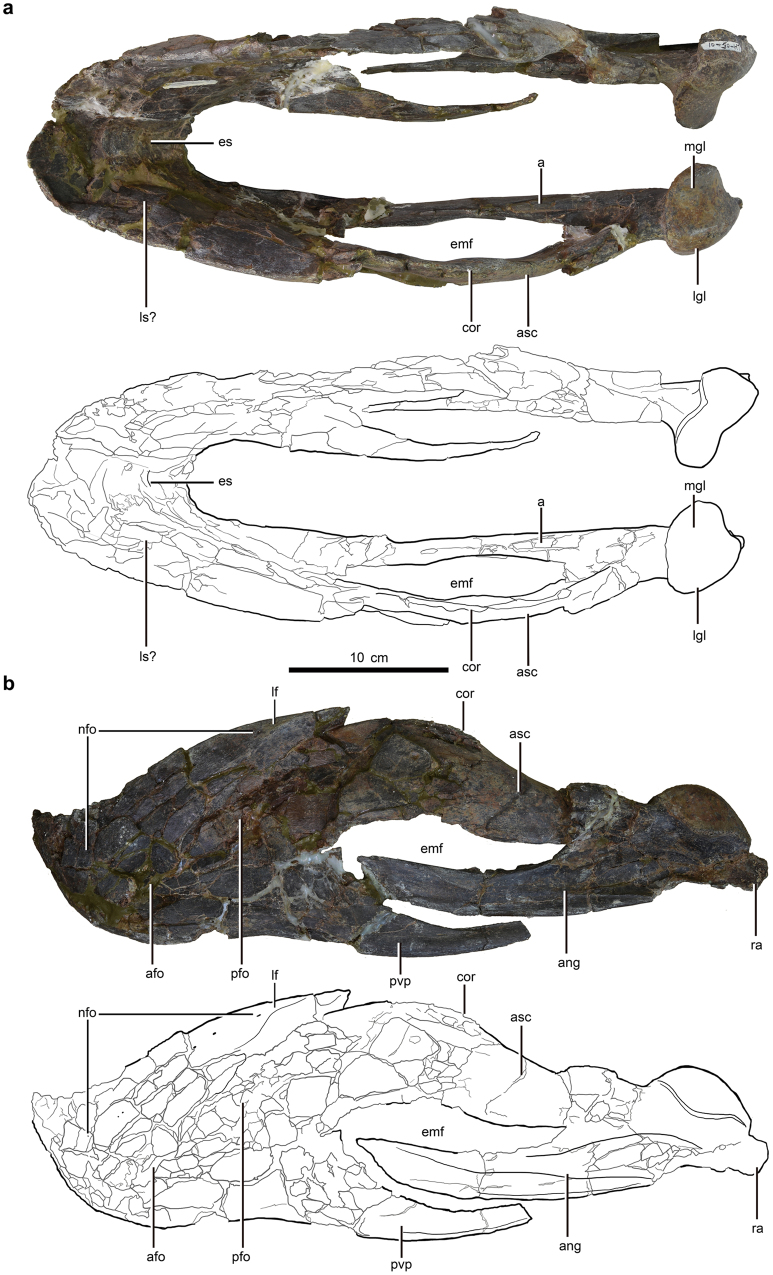
Mandible of *Gigantoraptor erlianensis* holotype LH V0011 in dorsal and left lateral view. (**a**) Dorsal view. (**b**) Left lateral view. Abbreviations: ang, angular; afo, anterior fossa; asc, articular-surangular-coronoid complex; cor, coronoid process; emf, external mandibular fenestra; es, extended shelf; nfo, nutrient foramina; lgl, lateral facet of articular glenoid; lf, lateral flange; ls, lingual triturating shelf; mgl, medial facet of articular glenoid; pfo, posterior fossa; pvp, posteroventral process of dentary; ra, retroarticular process. Scale is 10 cm.

**Figure 2:**
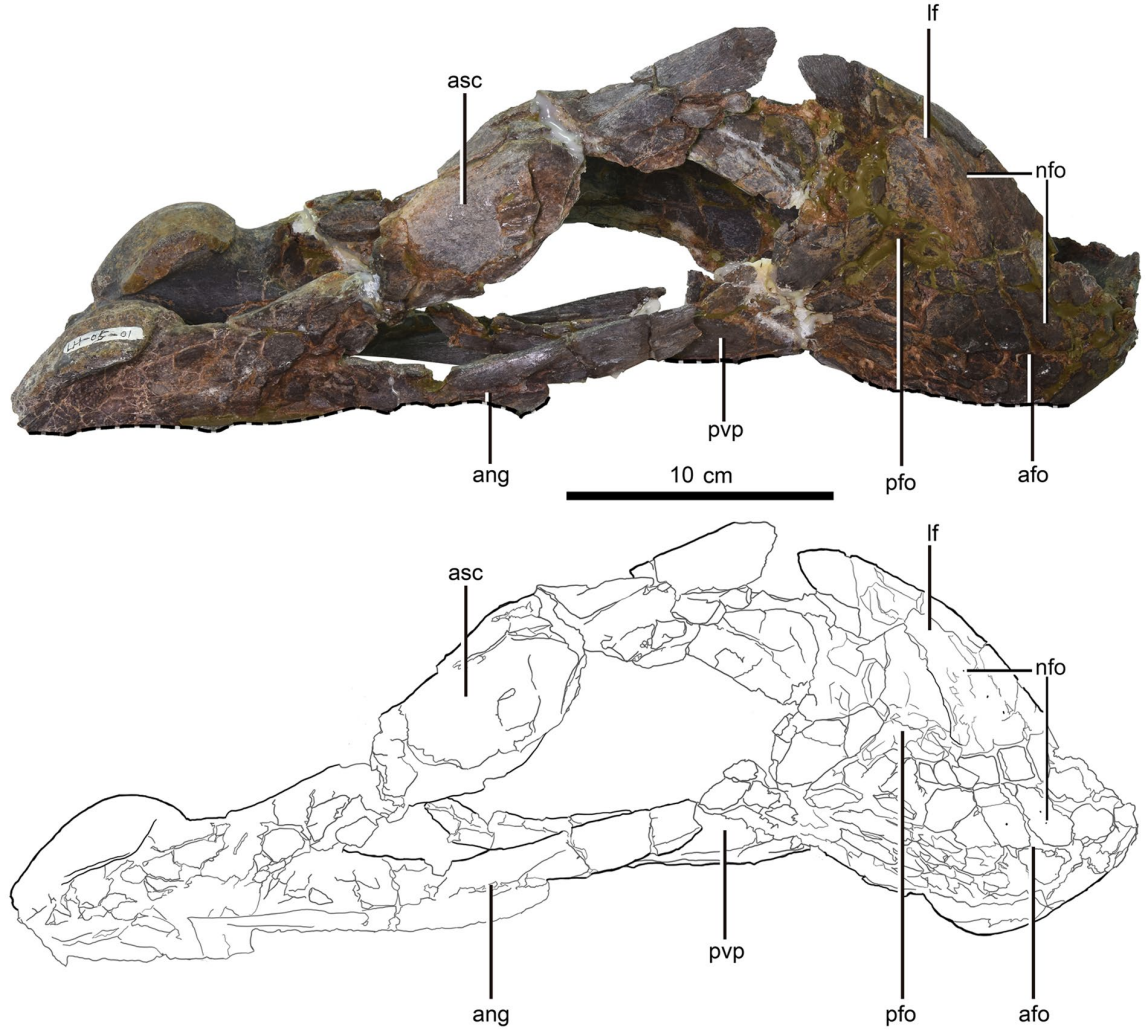
Mandible of *Gigantoraptor erlianensis* holotype LH V0011 in right lateral view. See Fig. 1 for abbreviations. Scale is 10 cm.

**Figure 3:**
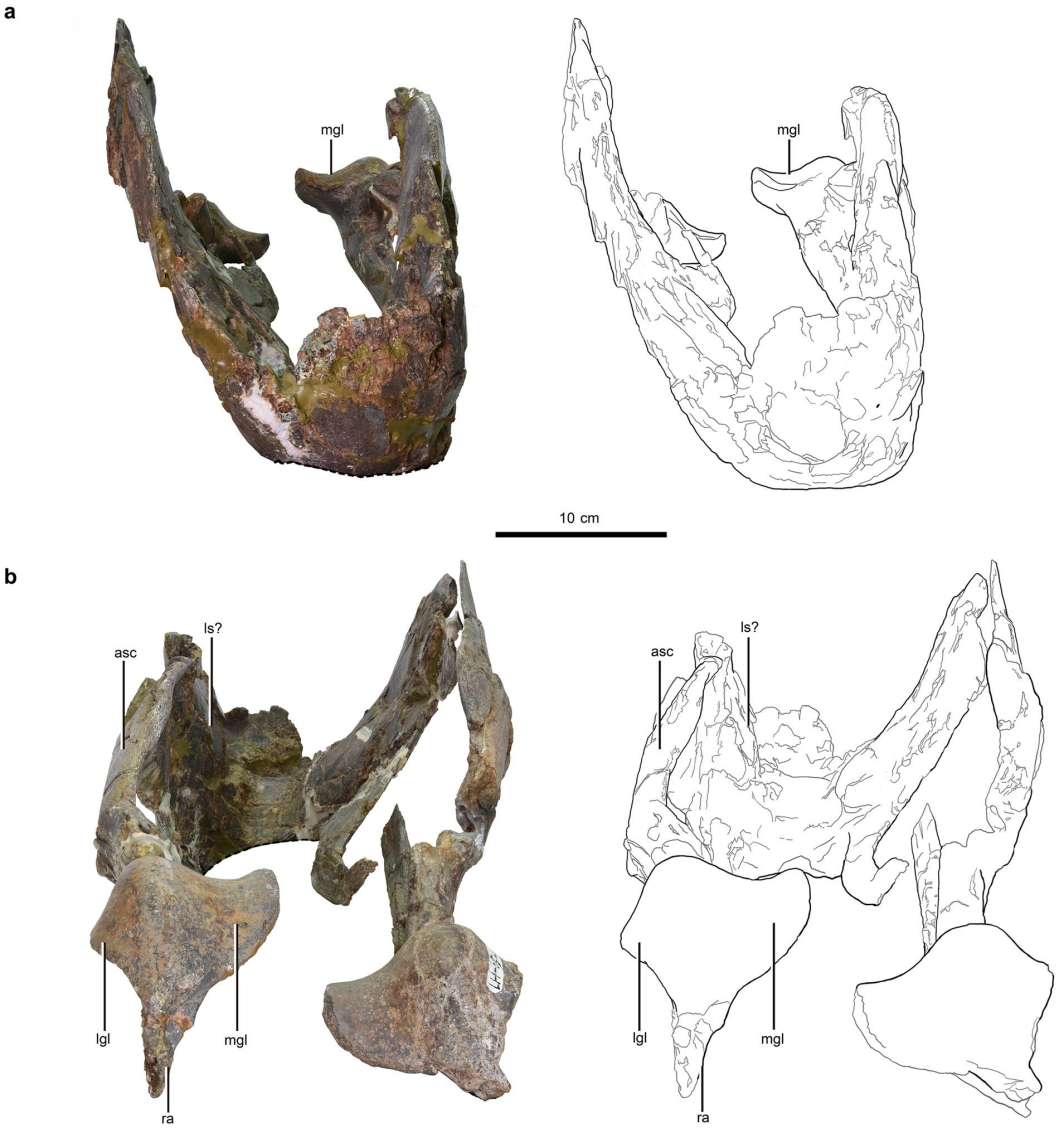
Mandible of *Gigantoraptor erlianensis* holotype LH V0011 in anterior and posterior view. (**a**) Anterior view. (**b**) Posterior view. See Fig. 1 for abbreviations. Scale is 10 cm.

The mandible of the holotype (LH V0011) is nearly complete (Figs 1–3), with the left portion being the best preserved (Fig. 1b) (see Supplementary Note online for Institutional Abbreviations and Material Description). The dentaries are completely preserved, but the right one is crushed and its posteroventral process is deflected medially. The angular and articular-surangular-coronoid complex (ASC complex) of the left dentary is well-preserved without much deformation (Fig. 1). However, the right ASC complex and right angular are broken and twisted to such an extent that their morphological interpretation is difficult (Figs 1–3). Both articular glenoid fossae are preserved (Figs 1–3). The retroarticular process is only preserved on the left articular (Figs 1 & 3).

### Dentary

The dentary is deep relative to the anteroposterior length of the mandible compared to other caenagnathids (see Supplementary Table S2). It is completely edentulous, as in oviraptorosaurs other than *Incisivosaurus gauthieri* ^1^, *Ningyuansaurus wangi* ^11^ and *Protarchaeopteryx robusta* ^2^. The occlusal edge of the dentary is sharp. The dentary symphysis is completely fused without any trace of a suture (Figs 1a & 3), as in *Incisivosaurus gauthieri* and other crownward caenagnathids (ref.^12^: character state 2 of character 73). It differs from oviraptorids like *Citipati osmolskae* ^24^ and *Yulong mini* ^25^ where a suture is discernible between the dentaries, but the degree of symphyseal fusion appears to increase with ontogeny^26^. The anteroventral surface of the symphysis is smooth (Fig. 3a) and it is strongly rounded in lateral view (Figs 1b & 2), as in *Leptorhynchos gaddisi* ^10^. Some oviraptorosaurs such as *Nemegtomaia barsboldi* and *Banji long* possess a ‘chin-like’ ventrally projecting process at the anteroventral margin of the dentary (ref.^27^: character state 1 of character 625), whereas this is absent in *Gigantoraptor* (Figs 1b & 2). The symphysis is slightly downturned in lateral view, unlike *Caenagnathus collinsi* which lacks a downturned portion^28^.

The lateral surfaces of the symphyseal region is recurved strongly towards the midline to form a U-shaped dentary in dorsal view (Fig. 1a), a characteristic of all oviraptorosaurs with the possible exception of *Luoyanggia liudianensis* ^29^. An extended symphyseal shelf (Fig. 1a) is present in *Gigantoraptor* as in other oviraptorosaurs except *Luoyanggia liudianensis* ^29^. Symphyseal ridges arranged almost perpendicular to the dorsal surface are absent, as in stemward oviraptorosaurs and all known oviraptorids. However, they are present in some caenagnathids which include *Caenagnathus collinsi* ^28^, *Caenagnathasia martinsoni* ^28,30^, *Leptorhynchos gaddisi* ^10^, *Leptorhynchos elegans* ^10^, *Chirostenotes pergracilis* ^9^ and *Anzu wyliei* ^15^.

In dorsal view, a possible lingual triturating shelf is indicated by a slight bulge in the medial surface of the dentary, although this surface is still quite flat (Figs 1a & 3b). This differs from caenagnathids more crownward than *Gigantoraptor* where the lingual shelf is prominent and well-developed medially in dorsal view. In stemward oviraptorosaurs like *Incisivosaurus gauthieri* ^1^ and the stemward caenagnathid *Microvenator celer* ^31^, this shelf is absent. This suggests that the weakly developed lingual shelf in *Gigantoraptor* is possibly an intermediate stage in the evolution of the lingual shelf in caenagnathid oviraptorosaurs. In specimens possessing the lingual shelf, the shelf is bound by the lingual ridge and occlusal grooves are present on the shelf ^9,10,15,28^. However, these features are absent in *Gigantoraptor*’s mandible. In oviraptorids, no lingual triturating shelf, lingual ridge or occlusal grooves have been noted in known specimens. The inner portion of *Gigantoraptor*’s dentary is therefore arguably more similar to oviraptorids than to crownward caenagnathids in general morphology.

**Figure 4:**
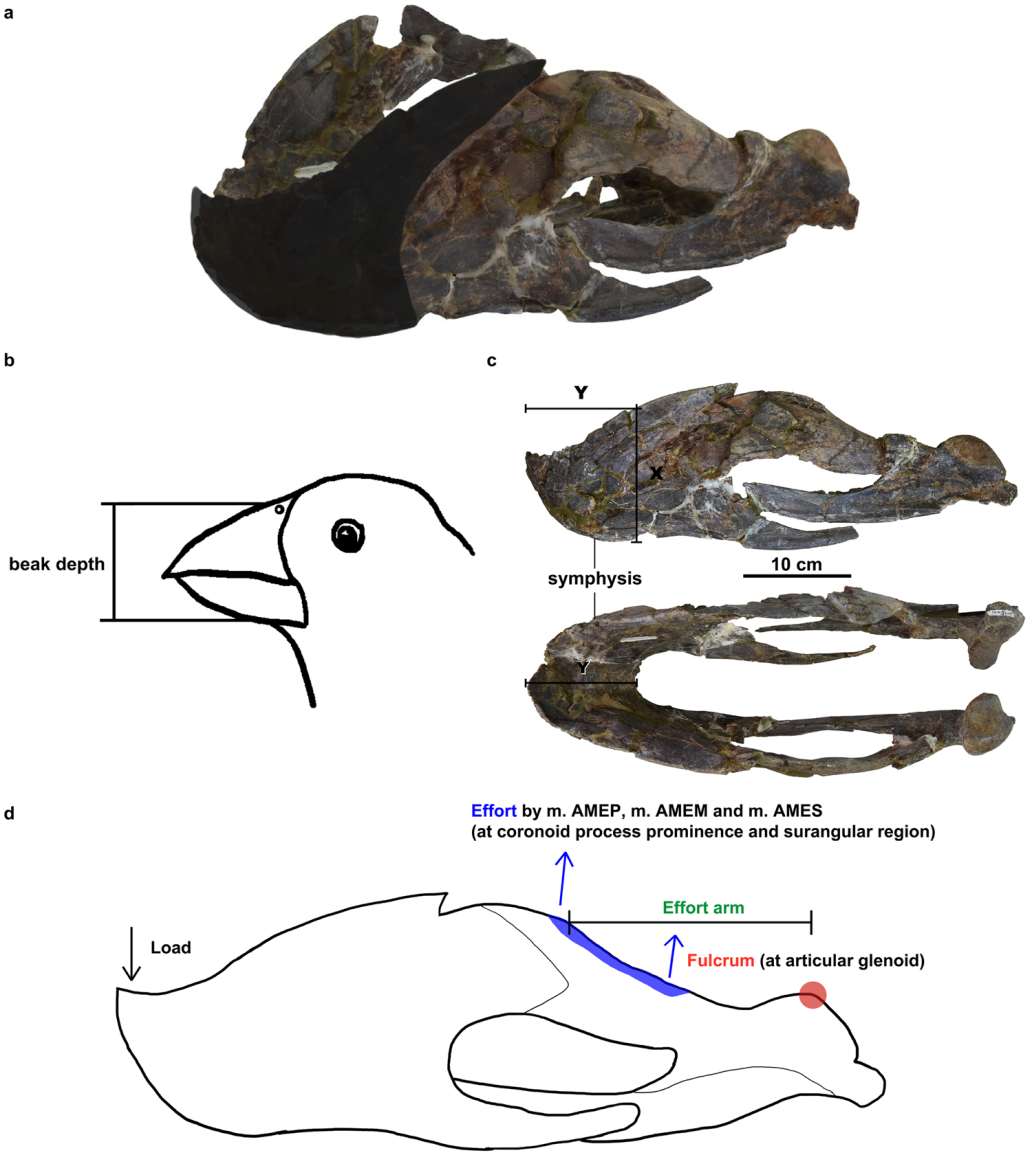
(**a**) Reconstructed rhamphotheca of *Gigantoraptor erlianensis* (LH V0011). (**b**) Schematic diagram showing how beak depth is usually measured in modern birds. Beak depth typically refers to the vertical depth measured at the anterior tip of the nostril perpendicular to the length of the beak and includes both upper and lower beaks^39–42^. (**c**) Relative beak depth measurement of *Gigantoraptor*’s mandible (LH V0011). X refers to the height behind the symphysis and Y refers to the anteroposterior length of the symphysis. respectively. Scale is 10 cm. (**d**) Jaw-closing system of oviraptorosaurs. Schematic diagram of *Gigantoraptor erlianensis* (LH V0011) in left lateral view showing the jaw-closing system relating to m. adductor mandibulae externus profundus (m. AMEP), m. adductor mandibulae externus medialis (m. AMEM) and m. adductor mandibulae externus superficialis (m. AMES).

A lateral flange is present on the lateral surface of the dentary (Figs 1b & 2), as in *Anzu wyliei* ^15^ and the Bayn Shire caenagnathid MPC-D 107/17^32^. This feature has not been observed in oviraptorids. Unlike *Anzu wyliei* ^15^ but similar to MPC-D 107/17^32^, the lateral flange does not extend anteriorly over the symphyseal region in *Gigantoraptor*. It extends posterodorsally above the posterior fossa with an angle of ~45 degrees relative to the ventral margin of the dentary. The posterodorsal end of the lateral flange forms a ‘prominent protrusion’ over the dorsal margin of the posterior extension of the dentary (Fig. 1b,c). This protrusion is absent in *Anzu wyliei* (ref.^15^: Fig. 3b), but its presence in MPC-D 107/17 is uncertain since this portion of the specimen is not preserved (ref.^32^: Fig. 4b).

Two fossae are present on the lateral surface of the dentary: the anterior fossa is located slightly nearer to the ventral margin than the dorsal margin, while the posterior fossa occupies a higher position ventral to the lateral flange (Figs 1b & 2). No pneumatopores are present, unlike in some caenagnathids like *Chirostenotes pergracilis* ^9^ and *Caenagnathus collinsi* ^28^ which have one or two pneumatopores close to the anterior margin of the external mandibular fenestra. As in other oviraptorosaurs, distinct nutrient foramina are present on the lateral surface of the dentary (ref.^27^: character state 1 of character 69).

In lateral view, the dorsal margin of the dentary is highly convex (Figs 1b & 2). The dentary diverges into dorsal and ventral rami posteriorly and forms the anterior margin of the external mandibular fenestra (Fig. 1b). The posterodorsal process extends about halfway across the fenestra (Fig. 1b), unlike in *Incisivosaurus gauthieri* and *Apatoraptor pennatus* (ref.^12^: Fig. 4) where the process stops above the anterior margin of the fenestra. The fenestra has an oval shape and is elongated anteroposteriorly (Fig. 1b), as in stemward oviraptorosaurs (*Incisivosaurus gauthieri* and *Caudipteryx zoui*) and in other caenagnathids (ref.^12^: character state 0 of character 170).

### Angular

The angular is tightly sutured with the ASC complex (Figs 1b & 2). It is taller dorsoventrally than wide mediolaterally and forms the ventral margin of the external mandibular fenestra (Figs 1b & 2). The angular extends anteriorly and bears a lateral elongated depression that forms the articular facet for the posteroventral process of the dentary. It bows outward along the ventral margin to form a ventral flange, visible in ventral view. It is presumed that the ventral flange lay below the posteroventral process of the dentary before the dentary was deformed, so this feature is probably not unique and diagnostic of *Apatoraptor pennatus* ^12^. However, the ventral flange of both specimens extends posteriorly vertically below the coronoid process prominence to nearly the posterior end of the mandible.

### Articular-surangular-coronoid (ASC) complex

The articular, surangular and coronoid of the mandible are ossified into a single unit called the articular-surangular-coronoid (ASC) complex, which was first reported in caenagnathids^28^. The ASC complex extends anteriorly over half the length of the external mandibular fenestra (Fig. 1b). The coronoid process prominence is dorsoventrally low (Fig. 1b), and distinct from those of oviraptorids^24,33^ and some crownward caenagnathids (e.g. *Caenagnathus collinsi* ^28^: Fig. 1) where the prominence is dorsoventrally high and hooked medially. In dorsal view, the surangular bulges laterally (Fig. 1a) whilst in medial view it forms the majority of the dorsal margin of the intramandibular fossa. No surangular foramen is present, unlike in *Banji long* ^34^ where three surangular foramina are preserved anterior to the articular region.

The articular glenoid is positioned strongly ventral to the dorsal margin of the dentary, as in other oviraptorosaurs (ref.^27^: character state 2 of character 623). In lateral view, it is dorsally convex, as in other caenagnathids and all oviraptorids (Figs 1b & 2). The shape of *Gigantoraptor*’s articular glenoid is similar to that of *Chirostenotes pergracilis* (ref.^9^: Fig. 1) and *Anzu wyliei* (ref.^15^: Fig. 3S), but is more dorsally convex than that of *Caenagnathus collinsi* in lateral view (ref.^35^: Fig. 1a). The mediolateral width of the lateral facet of the articular glenoid is narrower than its medial facet with a ratio of ~5:9 (see Supplementary Table S1). The medial facet is dorsally concave in posterior view (Fig. 3b) and forms a ‘bowl-shaped’ surface. In contrast, the lateral facet of the articular glenoid is steeply inclined, lacking a concave surface similar to the medial facet.

The retroarticular process extends posteroventrally from the articular (Fig. 1b), as in most oviraptorosaurs more crownward than *Avimimus portentosus* with the exception of *Apatoraptor pennatus* where the process points posterolaterally (ref.^12^: character state 1 for character 198). The retroarticular process is dorsoventrally taller than wide mediolaterally, similar to *Anzu wyliei* ^15^, but unlike in more crownward caenagnathids where the process is similar in height and width (ref.^12^: character state 1 of character 224). In oviraptorids, the process is either similar in width and height or its height is shorter than its width. In posterior view, the retroarticular process is slender and becomes narrower as it extends posteriorly (Fig. 3b). This condition contrasts with that of *Nemegtomaia barsboldi* and possibly *Citipati osmolskae* (ref.^24^: Fig. 10) where the retroarticular process is wide and makes up a flat surface.

### Ancestral state reconstruction of oviraptorosaur mandibular characteristics

**Figure 5:**
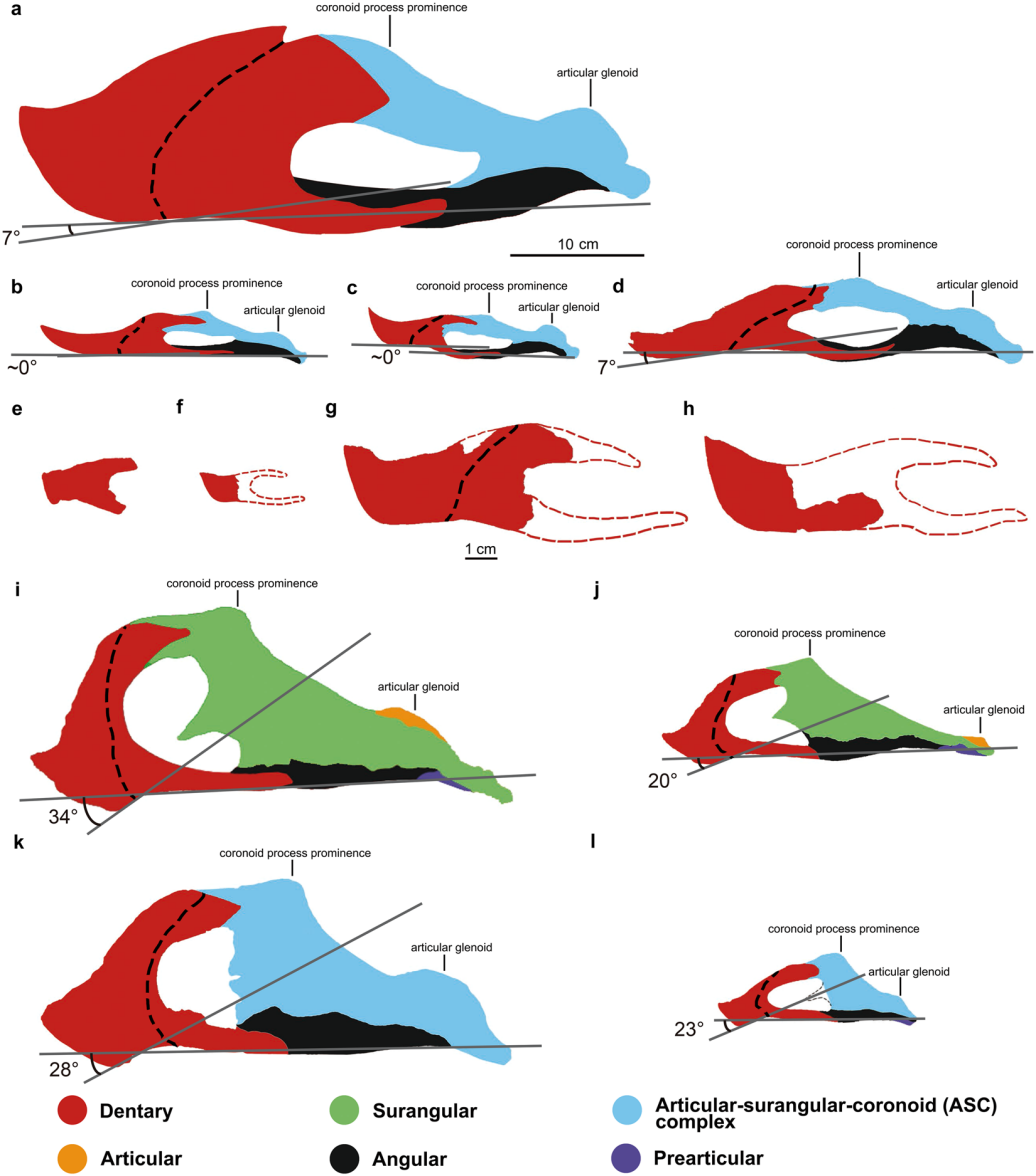
Caenagnathid and oviraptorid mandibles in lateral view showing reconstructed rhamphotheca extent and how degree of symphysis deflection was measured. Dotted line indicates the posterior extent of rhamphotheca. (**a**) *Gigantoraptor erlianensis* (qualitative reconstruction of LH V0011); (**b**) *Caenagnathus collinsi* (qualitative reconstruction of CMN 8776; modified from^28^); (**c**) *Chirostenotes pergracilis* (qualitative reconstruction of TMP 2001.12.12; modified from^9^); (**d**) *Anzu wyliei* (qualitative reconstruction of CM 78000; modified from^15^) (**e**) *Microvenator celer* (AMNH 3041; modified from^31^); (**f**) *Caenagnathasia martinsoni* (qualitative reconstruction of CMGP 401/12457; modified from^28^); (**g**) *Leptorhynchos elegans* (qualitative reconstruction of TMP 1992.36.390; modified from^10^); (**h**) *Leptorhynchos gaddisi* (qualitative reconstruction of TMM 45920–1; modified from^10^); (**i**) *Citipati osmolskae* (reconstruction of IGM 100/978; modified from^24^); (**j**) *Khaan mckennai* (reconstruction of IGM 100/973; modified from^88^); (**k**) *Nemegtomaia barsboldi* (reconstruction of GIN10012112; modified from^33^) and (**l**) *Yulong mini* (reconstruction of *Yulong mini*; modified from^25^). Scale is 10 cm in a-d; 1 cm in e-l.

The possession of a beak has been inferred in various potentially herbivorous non-avialan theropods, including oviraptorosaurs^5^. In addition to the anterior bony portion of the maxilla and mandible, a beak consists of an outer covering called the rhamphotheca. Reconstruction of the beak in non-avialan theropods largely relies on osteological correlates, as direct preservation of the rhamphotheca is rare and is only known in ornithomimosaur specimens so far^36,37^. In *Gigantoraptor*, the nutrient foramina and the depression (‘posterior fossa’) observed on the lateral surfaces of both dentary bones are likely to indicate the posterior extent of its rhamphotheca (Figs 1b & 2), as suggested for the therizinosaur theropod *Erlikosaurus andrewsi* ^36^ based on comparisons with modern birds^38^ and ornithomimosaur theropods^37^. The posterior-most foramen is located near the posterodorsal end of the lateral flange (Fig. 1b: left lateral view) and the ‘posterior fossa’/depression is located ventral to the lateral flange. As in^38^, a similar lateral depression on the dentary of the dusky parrot (*Pionus fuscus*) appears to trace the posterior extent of the rhamphotheca. Thus, it is likely that the rhamphotheca covering the mandible of *Gigantoraptor* extends posteriorly until meeting the posterior fossa (Fig. 4a). Regarding the dorsal extent of the rhamphotheca, we assume that it closely resembles the morphology of the symphyseal region, as suggested in the therizinosaur *Erlikosaurus andrewsi* ^36^. Figure 5 shows reconstructions of the posterior extent of the rhamphotheca covering the mandible of other oviraptorosaurs based on the same reasoning.

Relative beak depth varies within caenagnathids and some of them are considered to have a ‘deep beak morphology’^9,12^, although previous studies did not define ‘beak depth’ precisely. In ornithology, beak depth usually refers to the vertical depth measured at the anterior tip of the nostril perpendicular to the length of the beak, including both upper and lower beaks^39–42^ (Fig. 4b). However, this method is difficult to apply to fossil beaked animals as complete preservation of both beaks can be rare. A number of caenagnathid lower jaws are known^9,10,12,15,17,28,31,32,43,44^ (Fig. 5), whereas only *Anzu wyliei* ^15^ and possibly *Chirostenotes pergracilis* preserve a skull and mandible.

To facilitate future comparative work, a relative beak depth ratio for the mandible of oviraptorosaurs is proposed. Relative beak depth (X/Y) is the height behind the symphysis (X) relative to the anteroposterior/shortest length of the symphysis (Y) (Fig. 4c). Using symphyseal length and height behind the symphysis allows us to keep the metric to the mandible. Long bones like the femur may not be preserved in association with the mandible to account for size variation among different specimens: the relative beak depth of eleven caenagnathid mandibles were measured (see Supplementary Table S3) but only three of the specimens were associated with a femur (*Microvenator celer* (AMNH 3041), *Gigantoraptor erlianensis* (LH V0011) and *Anzu wyliei* (CM 78000). The symphysis is chosen as a reference point because the height behind the symphysis typically matches the vertical depth measured at the anterior tip of the nostril (“real beak depth”) in modern birds. Even for birds with longer beaks like gulls, the beak depth is usually measured at the gonydeal angle (the posterior tip of symphysis)^45,46^, which is actually the same as the height behind the symphysis.

**Figure 6:**
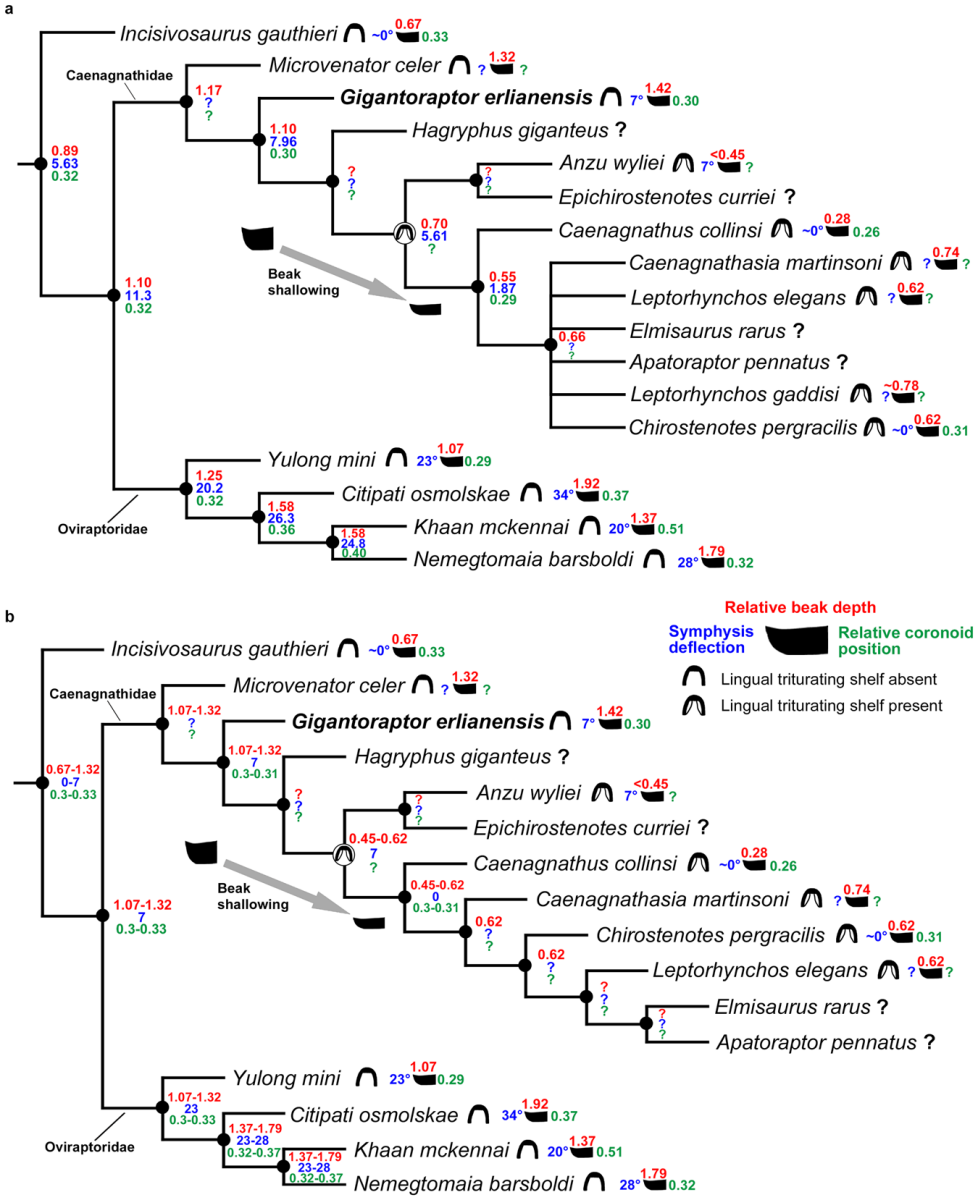
Ancestral state reconstruction of relative beak depth, degree of symphysis deflection, relative position of coronoid process prominence and the presence/absence of the lingual triturating shelf across Oviraptorosauria. (**a**) Analysis using the strict consensus tree of Funston & Currie^12^. (**b**) Analysis using a fully resolved topology that excludes *Leptorhynchos gaddisi* ^12^. See Methods.

When measuring the anteroposterior length of the symphysis, the orientation of the mandible should be standardized to minimise measurement inconsistency. Unfortunately, there is not a standard method to control skull orientation or define the horizon when beak length is measured in ornithology (or in other modern animals)^42,47^. Zanno *et al*.^48^ quantified the ventral deflection of the dentary in the therizinosaurs *Segnosaurus galbinensis* and *Erlikosaurus andrewsi* by measuring the angle between the ‘horizon’ – a ‘best fit’ of the ventral margin of the mandible (ref.^48^: Fig. 6) – and the orientation of the downturned symphyseal region. Here we adopt this method for standardising the orientation of the mandible for measurement purposes.

Based on this method, relative beak depth was calculated for caenagnathid and selected oviraptorid mandibles and for *Incisivosaurus gauthieri* (see Supplementary Table S3). The relative beak depth of *Gigantoraptor* is ~1.42, which is deeper than other caenagnathid beaks (see Supplementary Table S3).

To reconstruct the evolution of relative beak depth along the caenagnathid lineage in order to understand its implications, parsimony-based ancestral state reconstructions were performed using the strict consensus tree topology of Funston & Currie^12^ (Fig. 6; see Methods). The evolution of the lingual triturating shelf was also reconstructed because it has been suggested to be functionally similar to the dentary table of the dicynodont *Diictodon feliceps*, which provides shearing edges to improve shearing effectiveness^9^. In this way, the evolution of the lingual triturating shelf could reveal possible changes in caenagnathid shearing ability. In addition to these traits, we also reconstructed the position of the coronoid process prominence and the degree of symphysis deflection across Oviraptorosauria (Fig. 6; see methods).

This analysis reveals a shallowing of the beak and the development of the lingual triturating shelf along the caenagnathid lineage. The node shared by *Microvenator* and *Apatoraptor* shows a beak depth of 1.17 and the node shared by *Gigantoraptor* and *Apatoraptor* shows a slightly smaller value of 1.10. Successive nodes show similar lower values for beak depth between 0.55–0.70 (Fig. 6a). In the analysis using the fully resolved tree topology that excludes *Leptorhynchos gaddisi* (Fig. 6b), the node shared by *Microventor* and *Apatoraptor* and the node shared by *Gigantoraptor* and *Apatoraptor* have the same reconstructed values (between 1.07 and 1.32). Successive nodes have reconstructed values between 0.45 and 0.62 (Fig. 6b). Funston & Currie^12^ inferred a ‘deep beak morphology’ for *Apatoraptor* based on the preserved portion of the mandible. If a relative beak depth of 0.62 is used for *Apatoraptor* based on *Leptorhynchos* (its closest relative preserving a complete beak), the node shared by *Apatoraptor* and *Leptorhynchos* (the most crownward caenagnathids) also has a value of 0.62 in the fully resolved tree (Fig. 6b). This further supports a shallowing of the beak between the node shared by *Microvenator* and *Apatoraptor* and the node shared by *Anzu* and *Apatoraptor*. It also supports relatively constant beak depth along the remaining nodes of the caenagnathid lineage. The relative position of coronoid process prominence does not vary significantly along the caenagnathid lineage, with reconstructed values of 0.29–0.31 for all the nodes in both trees (Fig. 6). The oviraptorid lineage has nodal values of 0.3–0.4, which are slightly larger than those of the caenagnathid lineage in general (Fig. 6). For the degree of symphysis deflection, in the less resolved tree, the reconstructed values decrease slightly along the caenagnathid lineage (Fig. 6a): decreasing from 7.96° at the node shared by *Gigantoraptor* and *Apatoraptor* to 1.87° at the node shared by *Apatoraptor* and *Caenagnathus* (Fig. 6a). A similar decrease is also observed along the caenagnathid lineage in the resolved tree (Fig. 6b). In both trees, the oviraptorid lineage has significantly larger nodal values (20.2–28°) than those of the caenagnathid lineage (Fig. 6).

## Discussion

Associations between beak shape and diet in living birds are a helpful tool for understanding the same traits in the extinct beaked bird-like oviraptorosaur dinosaurs. The functional significance of beak depth variation in some modern birds has been studied previously, although only limited to a few species. In the medium ground finch (*Geospiza fortis*), the depth of the beak is positively correlated with bite force^49^. During drought periods, these birds tend to maintain deeper and narrower beaks to process harder and less abundant seeds^50^. Parrots (Psittaciformes) with generalist lifestyles have a seed-based diet^51^ and also possess a deep beak. In Oviraptorosauria, the beak of *Leptorhynchos* (Caenagnathidae) has been suggested to be potentially capable of processing tough and fibrous plants, although the reasons for this were not given^10^. A relatively deep mandible in ankylosaurs may have also been related to the mastication of tough materials^52^. *Gigantoraptor* has the deepest beak among known caenagnathids (see Supplementary Table S3), so the trend of initial beak shallowing and then its maintenance along the caenagnathid lineage (Fig. 6), might indicate a reduction in the capacity to process harder food (as it does in modern birds), possibly in relation to a dietary change. However, this hypothesis remains tentative in the absence of quantitative musculoskeletal modelling^23,53^ and the absence of other well-preserved stemward caenagnathid mandibles (Fig. 5). There is also the possibility that allometric growth might be responsible for observed differences in beak shape, but this requires further specimen discovery to test.

*Gigantoraptor* had a comparatively spacious buccal cavity compared to more crownward caenagnathids due to a deeper dentary and a lack of a lingual triturating shelf. This may have meant that it had a comparatively larger and more flexible tongue. In Psittaciformes, a large muscular tongue is present inside their deep dentary, unlike the typically narrow and thin tongues of other birds^54^. Psittaciformes use their tongue to position nuts and seeds in their mouth, including to crush food between their upper and lower beaks^54^. The large, mobile tongues of *Sphenodon* and iguanian lizards are also capable of repositioning food materials and assisting in swallowing^55,56^. If *Gigantoraptor* had a proportionally larger and more mobile tongue this may have been similarly helpful in positioning food for processing, potentially improving feeding efficiency. This trait might therefore have become less important in subsequent caenagnathids, which probably had comparatively smaller and less mobile tongues in their comparatively smaller buccal cavities.

The lingual surface of *Gigantoraptor*’s dentary is nearly vertical, different from more crownward caenagnathids where the lingual triturating shelf is present. The triturating function of the lingual shelf was first proposed by Longrich *et al*.^10^ based on its similarity with that of typical tortoises (*Geochelone*). A lingual triturating shelf is present in *Chirostenotes pergracilis*, and has been suggested to act as shearing edges to improve shearing ability^9^. Thus, the evolution of the lingual triturating shelf in crownward caenagnathids (Fig. 6) appears to suggest increased specialization in the shearing of food materials along the caenagnathid lineage.

Two fossae are present on the lateral surface of the dentary of *Gigantoraptor*. A previous skull reconstruction of the ornithischian dinosaur *Psittacosaurus gobiensis* ^57^ has placed the m. adductor mandibulae externus ventralis (m. AMEV) on a similar depression on the lateral surface of the dentary, although the inference of this muscle is not well-supported by phylogenetic bracketing^58^. If the m. AMEV attached on any of the fossae on the lateral surface of the dentary of *Gigantoraptor*, this may indicate that *Gigantoraptor* gained extra bite force from this muscle as in *Psittacosaurus luiiatunesis* ^58^. In such a scenario the m. AMEV may have functionally resembled the pseudomasseter muscle that inserts onto the lateral surface of the mandible of modern parrots^59^, acting as an important mechanism to produce a strong bite^60^. In Caenagnathidae, the two most stemward members *Microvenator* ^31^ and *Gigantoraptor* have a lateral depression immediately anterior to the external mandibular fenestra while more crownward members do not (ref.^12^: character state 0 of character 217). Some oviraptorids are also reported to have such a depression, such as *Nankangia jiangxiensis*, *Ganzhousaurus nankangensis*, *Nemegtomaia barsboldi*, *Heyuannia huangi* (ref.^12^: character state 0 of character 217) and *Tongtianlong limosus* ^18^. However, no clear evolutionary trend is identified in oviraptorids, unlike caenagnathids. If the presence of a lateral depression on the dentary indicates the insertion of m. AMEV in oviraptorosaurs, this could potentially suggest a decreased ability in bite force generation in caenagnathids more crownward than *Gigantoraptor*. However, this muscle inference remains tentative but deserves further investigation.

The coronoid process prominence in *Gigantoraptor* is located at a similar position to other caenagnathids. It is suggested to be the attachment site for m. adductor mandibulae externus profundus (m. AMEP), a jaw closing muscle of non-avialan dinosaurs^61^ that has been modelled in recent musculoskeletal cranial reconstructions of *Psittacosaurus* ^58^ and selected therizinosaur and ornithomimosaur theropods that also evolved dietary specializations^53,62^. Other adductor mandibulae externus muscles, including m. adductor mandibulae externus superficialis (m. AMES) and m. adductor mandibulae externus medialis (m. AMEM), have been inferred to attach onto the dorsolateral and dorsomedial surface of the surangular or on the coronoid process prominence in these studies^53,58,61,62^. In the jaw-closing system related to the adductor mandibulae externus group, the jaw joint (articular glenoid) acts as a fulcrum and the coronoid process prominence and surangular region are the points where muscle effort is acted on (Fig. 4d). In general, a more anteriorly-positioned coronoid process prominence and surangular region (longer effort arm) would provide a greater mechanical advantage, which generates a larger bite force. The distance between the coronoid process and articular glenoid in *Gigantoraptor* is slightly more than one-third of the anteroposterior length of the mandible (see Supplementary Table S1). This is similar to the ratio in *Chirostenotes pergracilis* ^9^ and *Caenagnathus collinsi* based on estimation from figures in^28^ (see Supplementary Table S4). The mechanical advantages of the jaw-closing systems of these caenagnathids are also similar (0.30–0.35) (see Supplementary Table S4). The coronoid process prominence of *Gigantoraptor* appears to be positioned vertically lower compared to other caenagnathids (Fig. 5). A dorsoventrally taller coronoid process is correlated with a larger bite force in lacertid lizards by having a larger adductor muscle attachment site^63^. The relationship between a dorsoventrally tall coronoid process prominence and large bite force is also observed in more distantly related vertebrates, including phyllostomid bats^64^ and moray eels (muraenid fish)^65^. Although the beak of *Gigantoraptor* is significantly deeper than crownward caenagnathid beaks, the coronoid process prominence is not as dorsoventrally tall as we might expect based on modern animals with stronger bites and harder food diets^63–65^.

The dorsally-convex articular glenoid of *Gigantoraptor* is likely to indicate propalinal jaw movement during food processing. The articular regions of dicynodonts^66^, caenagnathids and oviraptorids (ref.^12^: character state 1 of character 91) all have a convex articular glenoid that was suggested to have allowed anteroposterior lower jaw movement^9,24,67^. However, understanding oviraptorosaur jaw mechanics from another extinct animal group is not ideal owing to the lack of preserved soft tissues. Recently, Longrich *et al*.^10^ suspected that a similar articular glenoid is present in *Gallus domesticus*, but the associated jaw mechanics were not studied in detail. We note that *Sphenodon* possesses an articular region which closely resembles that of *Gigantoraptor* and crownward caenagnathids as well as of oviraptorids. An in-depth study on the feeding mechanics of *Sphenodon* reveals how they achieve propalinal jaw movement^68^. With respect to a linear motion guide system, the articular region functions similarly as the ‘rail’ and the quadrate condyle functions as the ‘slide’. With such a configuration, the mandible can slide forward relative to the maxilla after occlusion for shearing of food^68^. The detailed mechanics of the *Sphenodon* functional analogue strengthens the basis for suggesting propalinal jaw movement and shearing in *Gigantoraptor* and other caenagnathids with a similar articular region, including *Chirostenotes pergracilis* ^9^, *Anzu wyliei* ^15^ and *Caenagnathus collinsi* ^28^. However, this hypothesis would greatly benefit from future quantitative musculoskeletal modelling, which could reveal novel subtleties in caenagnathid jaw function^23,53^.

Caenagnathids and oviraptorids show a number of differences in their mandible morphologies^69,70^ (Fig. 5). Longrich *et al*.^10^ noticed that caenagnathid mandibles are generally longer and shallower when compared to those of oviraptorids, which they suggested indicates different feeding strategy. It appears that the mandibles of caenagnathids are generally more adapted to shearing actions, whereas those of oviraptorids generally seem to favour the production of a stronger bite, potentially related to other feeding styles e.g. crushing action. Oviraptorids usually possess a more downturned symphyseal region of the dentary (~ <10°) in comparison to caenagnathids (~20–35°) (regarded as a herbivorous trait by Zanno & Makovicky;^5^ see Figs 5, 6 and Supplementary Table S5). In finches, a more decurved beak is suggested to be linked to the production of a more powerful bite, which is probably an adaptation for granivory and/or probing^71,72^. Parrots display an overall anatomy which is likely to be adapted for effective crushing, including possession of a suborbital arch and a special jaw-closing muscle (m. pseudomasseter)^51^. If its unique downturned beak portion is also shown to be an adaptation for crushing, the similar beak morphology of oviraptorids^16^ could suggest the ability for a comparable feeding mode. The less downturned portion in caenagnathids may therefore suggest a lesser capability compared to oviraptorids for feeding behaviours involving strong bite forces e.g. crushing. Oviraptorids do not possess a lingual triturating shelf, whereas it is present in crownward caenagnathids for a more effective shearing action^9^ (Fig. 6). This indicates that shearing-related feeding was more strongly selected upon along the caenagnathid lineage compared to the oviraptorid lineage.

Oviraptorids generally have dorsoventrally taller coronoid process prominences than caenagnathids^69^ (Fig. 5). As discussed above, the coronoid process prominence and surangular region of oviraptorosaurs are likely to be the attachment site of the adductor mandibulae externus group and to be correlated with bite force (Fig. 4d). A large bite force was suggested in the oviraptorid *Yulong mini* based on the presence of a tall coronoid process prominence^25^ (Fig. 5l). The generally higher coronoid process prominences of oviraptorids compared to caenagnathids therefore suggests that oviraptorids were more capable of producing a stronger bite compared to caenagnathids. Oviraptorids may have also gained mechanical advantage (0.3–0.55) by having a slightly more anteriorly positioned coronoid process prominence than caenagnathids (0.3–0.35), a difference that is easy to miss without direct measurements (see Supplementary Table S4). The relative size of the medial mandibular fossa also appears to be larger in oviraptorids than in caenagnathids. The medial mandibular fossa is reconstructed as the attachment site for a number of muscles in dinosaurs, including m. adductor mandibulae externus posterior (m. AMP), m. pseudotemporalis superficialis (m. PSTs) and possibly m. pseudotemporalis profundus (m. PSTp)^53,61,62^. Due to the highly arched dentary, oviraptorids generally appear to have a larger medial mandibular fossa than caenagnathids (Fig. 5). As the attachment site for adductor and pseudotemporalis muscles in oviraptorosaurs, a larger fossa could potentially accommodate larger muscles producing a stronger jaw-closing action. Although the possibility that caenagnathids possess a medial mandibular fossa which is significantly deeper lateromedially than that of oviraptorids cannot be ruled out, it is likely that oviraptorids had relatively larger muscles in the medial mandibular fossa than caenagnathids, if their fossae show similar lateromedial depths. Oviraptorids also display a larger external mandibular fenestra than that of caenagnathids in general (Fig. 5). In some crocodylians, a smaller external mandibular fenestra was suggested to be associated with a less developed musculus intramandibularis (MI, a muscle which was hypothesized to be homologous with m. pseudotemporalis^73^)^74^. Although the presence of m. intramandibularis has not been confidently inferred in any dinosaurs^61^, if future discoveries support this inference or strengthen its homology with m. pseudotemporalis, a larger external mandibular fenestra in oviraptorids than caenagnathids may indicate a better capability for adduction. Together with their taller coronoid process prominences, oviraptorids are likely to have produced stronger bite forces than caenagnathids, which indicates a likely difference in their feeding styles (e.g. more crushing-related feeding activities in oviraptorids compared to caenagnathids).

The possession of a beak was linked to a shift from carnivory to herbivory in many coelurosaur lineages including Oviraptorosauria^5^, although the heavily worn teeth of the stemward oviraptorosaur *Incisivosaurus* still provides the most direct evidence of oviraptorosaurian herbivory to date. A downturned dentary is considered as one of the characteristics indicating the evolution of a beak and herbivory, and is suggested to be commonly possessed by oviraptorosaurs more crownward than *Incisivosaurus gauthieri* and *Protarchaeopteryx robusta* ^5^. Later discoveries show that this character is found in oviraptorids, *Gigantoraptor* and other caenagnathids, but not in *Caenagnathus collinsi* ^23^ and *Tongtianlong limosus* ^18^. Crownward oviraptorosaurs have experienced an exceptionally high rate of evolution in skull anatomy and attained a bizarre cranial form compared to other theropods^75^. It is likely that after a beak evolved in stemward oviraptorosaurs, its high plasticity allowed the diversification of beak forms in more crownward oviraptorosaurs without being limited by the constraints encountered in early beak evolution^5^.

The shape and size of the feeding apparatus in large herbivores is thought to be related to dietary selectivity. These relationships have been observed in modern ungulates^76^ and inferred in herbivorous dinosaurs^77^. Selective herbivores typically prefer to consume seeds, fruits or foliage, the most nutritious parts of a plant^78^. The narrow beak of ceratopsians is likely to indicate selective feeding^77,79^ whereas the wide beak of *Euoplocephalus* and the U-shaped beak morphology of Hadrosauridae may suggest a less selective one with reference to the feeding behavior of modern herbivorous mammals^77^. Based on beak morphology, members of Hadrosauridae are believed to be intermediate feeders which consume parts with variable nutritional quality (e.g. foliage, fruits, seeds and twigs)^77^. The oviraptorosaur beak region also displays variation that is possibly linked to diet selectivity. Based on the aforementioned association between beak shape and feeding selectivity, the more squarish beak of *Nemegtomaia barsboldi* and the U-shaped beak of *Gigantoraptor* may indicate a less selective diet than the narrower beak of *Leptorhynchos elegans*, if they had similar sizes. However, the mouth size of large herbivores also affects feeding selectivity, with a smaller size linked to higher selectivity, as observed in modern mammals^76,80^. A larger mouth has a wider oral margin compared to a smaller mouth even if they have similar shapes, such that the exclusion of less nutritious plant parts is limited in the larger one. Based on this reasoning, *Chirostenotes pergracilis* is likely to be more selective than *Gigantoraptor* because of its smaller beak (width = ~5 cm)^9^ compared to *Gigantoraptor*’s (width = ~10 cm), even though they both possess a U-shaped beak. Taking both the shape and size of beak into consideration, oviraptorosaurs which have a small narrow beak (e.g. *Leptorhynchos elegans*) are likely to have had a relatively high feeding selectivity. In contrast, *Gigantoraptor*’s exceptionally large U-shaped beak may potentially indicate one of the most non-selective diets among oviraptorosaurs.

As a stemward caenagnathid, *Gigantoraptor* has an expected intermediate mandibular morphology — its dentary is similar to oviraptorids, but its post-dentary region closely resembles those of crownward caenagnathids. It therefore appears to have been capable of shearing and crushing-related feeding but perhaps with less fluency than crownward caenagnathids and crownward oviraptorids, or perhaps it had a unique feeding style afforded by this unique mandible. Another factor that might have influenced *Gigantoraptor*’s diet and feeding style is the greater energy requirements of larger animals^81^. Larger animals usually intake food with lower quality since it is available in larger quantities and has a more stable supply^82,83^. However, it is unclear whether its digestive system was specialized to support this presumably large food intake, as in sauropodomorphs^84^. Whilst a large U-shaped beak could indicate less dietary selectivity, carnivory cannot be ruled out as a strong beak together with a propalinal jaw movement may have also permitted processing of meat, as in toothed *Sphenodon* ^68^. *Gigantoraptor* was deposited in a fluvial sequence so it appears to have lived in changeable surroundings where a more generalist feeding strategy could have been advantageous. However, whilst this and its mandibular features may appear to hint more at a generalist feeding strategy based on currently available evidence, future quantitative reconstructions of *Gigantoraptor*’s bite performance as well as geochemically-informed dietary inferences are needed to secure our understanding of the feeding habitats of this fascinating animal^23^.

## Conclusions

The mandible of *Gigantoraptor* was described in detail for the first time. Its beak has the greatest relative depth among caenagnathids, a parameter that decreases overall along this lineage. This parameter does not appear to be correlated with bite force in the same way as modern animals because *Gigantoraptor* has a coronoid process prominence that is relatively low rather than high and its height varies among caenagnathids. The possession of the lingual triturating shelf in caenagnathids more crownward than *Gigantoraptor* (a feature absent in oviraptorids) suggests an increased specialization towards shearing along the caenagnathid lineage, possibly related to a dietary shift. The dorsally convex articular region of *Gigantoraptor* and other oviraptorosaurs suggests that propalinal jaw movement was likely to be an important part of food processing based on its morphological convergence with that of *Sphenodon* as well as dicynodonts. In comparison, oviraptorids have more downturned beaks, dorsoventrally taller coronoid process prominences and larger medial mandibular fossae, suggesting specialization towards feeding styles that utilize a stronger bite force e.g. crushing-related feeding. Despite having an unusually large body size, *Gigantoraptor* displays an intermediate mandibular morphology which suggests rudimentary shearing and crushing-related feeding capabilities when compared to crownward caenagnathids and crownward oviraptorids or perhaps even a unique feeding style related to the energy needs of such a large animal. This study provides new data and functional analogues that reinforce suggestions that the two main oviraptorosaur lineages — Caenagnathidae and Oviraptoridae — had divergent feeding styles likely to be linked with divergent dietary preferences.

## Methods

The *Gigantoraptor erlianensis* holotype LH V0011 is housed at the Longhao Institute of Geology and Paleontology, Nei Mongol in accordance with local regulations and is available for scientific study. Standard comparative anatomy methods were used to study the specimens discussed in this paper.

The angles of deflection in our caenagnathid, oviraptorid and *Incisivosaurus* mandibles were measured based on a method used in Zanno *et al* ^48^. (see Fig. 5 & Supplementary Table S5). The ventral deflection of the dentaries of the therizinosaurs *Segnosaurus galbinensis* and *Erlikosaurus andrewsi* were quantified by measuring the angle between the horizontal line and line of deflection^48^. The horizontal line is a ‘best fit’ of the ventral margin of the mandible that does not take the downturned portion into account (ref.^48^: Fig. 6). The deflection line is drawn according to the ventral margin of the downturned symphyseal portion (ref.^48^: Fig. 6). The same method has been used to measure the slope of the ventral margin of the beak of finches^85^. The relative position of coronoid process prominence is defined as the anteroposterior length between coronoid process prominence and articular glenoid/total mandibular length.

Ancestral state reconstructions for the absence/presence of the lingual triturating shelf, relative beak depth, degree of symphysis deflection and relative position of coronoid process prominence were performed in the evolutionary analysis software *Mesquite 3*.*20* using the program’s ‘parsimony ancestral state reconstruction method’ and the tree topologies presented in Fig. 6 ^86,87^. For Fig. 6a, squared change parsimony was used to reconstruct relative beak depth owing to the presence of a polytomy in the tree topology, whereas in Fig. 6b linear parsimony was used instead because the tree topology was fully resolved.

### Data availability

The data reported in this paper are detailed in the main text and in the Supplementary Information.

## Electronic supplementary material

Supplementary Information
