# Supplementary material for: Functional anatomy of a giant toothless mandible from a bird-like dinosaur: Gigantoraptor and the evolution of the oviraptorosaurian jaw
Source: Sci Rep. 2017 Nov 24;7:16247. doi: 10.1038/s41598-017-15709-7 (PMC5701234; doi:10.1038/s41598-017-15709-7)
Supplement: Supplementary file 1 — Supplementary Information [file 41598_2017_15709_MOESM1_ESM.docx]

Functional anatomy of a giant toothless mandible from a bird-like dinosaur: *Gigantoraptor* and the evolution of the oviraptorosaurian jaw

Waisum Ma, Junyou Wang, Michael Pittman*, Qingwei Tan, Lin Tan, Bin Guo & Xing Xu*

*Corresponding authors. M.P. ([mpittman@hku.hk](mailto:mpittman@hku.hk)) or X.X. ([xingxu@vip.sina.com](mailto:xingxu@vip.sina.com)).

**Supplementary Information**

Content:

Supplementary Notes

Supplementary Tables S1-S5

Supplementary References

**Supplementary Note**

**Institutional Abbreviations**

CM, Carnegie Museum of Natural History, Pittsburgh, Pennsylvania, USA; CMN, Canadian Museum of Nature, Ottawa, Ontario, Canada; IVPP, Institute of Vertebrate Paleontology and Paleoanthropology, Chinese Academy of Sciences, Beijing, China; LH, Long Hao Institute of Geology and Paleontology, Department of Land Resources, Hohhot, Nei Mongol, China; MPC, Mongolian Paleontological Center, Mongolian Academy of Sciences, Ulaanbaatar, Mongolia; TMM, University of Texas Memorial Museum, Austin, Texas, USA; TMP, Royal Tyrrell Museum of Palaeontology, Drumheller, Alberta, Canada.

**Material Description**

**Systematic Palaeontology**

DINOSAURIA Owen, 1842

SAURISCHIA Seeley, 1888

THEROPODA Marsh, 1881

COELUROSAURIA Huene, 1914

MANIRAPTORA Gauthier, 1986

OVIRAPTOROSAURIA Barsbold, 1976

*GIGANTORAPTOR ERLIANENSIS* Xu *et al*., 2007

**Holotype:** LH V0011, a partially complete skeleton consisting of a nearly complete mandible and several postcranial bones (right scapula, forelimbs, presacral vertebrae, ilium, pubes, hindlimbs and caudal vertebrae).

**Locality and horizon:** LH V0011 was discovered in Saihangaobi (赛罕高毕), Sunitezuoqi (苏尼特左旗), Nei Mongol, China (Fig. 1) in the Upper Cretaceous Erlian (Iren Dabasu) Formation (二连大巴苏组).

LH V0011 was collected from the Erlian Formation which has a controversial age. Based on the vertebrate fauna recovered, the formation was suggested to be early Senonian [1] but later research on microfossils (ostracods, charophytes, pollen & palynomorphs) estimated the age to be middle Campanian to early Maastrichtian [2] and late Campanian to early Maastrichtian [3, 4]. Xing *et al.* [2] suggested the age could be further refined to the middle to late Campanian based on dinosaur faunal composition. However, we conservatively adopt a middle Campanian to early Maastrichtian age in respect of both the micro- and macrofossil evidence available.

**Additional osteological description**

The text below provides additional details of the mandible of *Gigantoraptor* that were not pertinent to the salient osteological description provided in the main text.

In lateral view, the anterodorsal tip of the dentary is upturned with an angle greater than 45 degrees (~60 degrees) relative to the ventral margin of the symphysis (Fig. 2B). Some crownward caenagnathids such as *Caenagnathus collinsi* [5] and *Anzu wyliei* [6] have a beak that is less upturned than those of other caenagnathids.

Two fossae are present on the lateral sides of the dentary. The diameters of the anterior and posterior fossae are estimated to be ~3cm and ~2cm respectively, with the former deeper than the latter. Most caenagnathids, if not all, are reported to have deep fossae or lateral depressions on the lateral surface of dentary. However, the fossae of other caenagnathids are present at relatively more posterior positions than the anterior fossae in *Gigantoraptor*. They probably correspond topologically to the posterior fossa in *Gigantoraptor*.

Foramina are observed on the lateral sides of the dentary. At least six foramina are arranged in a row approximately parallel to the ventral margin of the left dentary. A few more foramina are also scattered across the lateral surface, although they do not appear to form any row, unlike caenagnathids such as *Chirostenotes pergracilis* [7] and MPC-D 107/17 [8] which appears to have three rows of foramina. However, it may be possible that rows of foramina have been damaged or destroyed by the deformation experienced by the *Gigantoraptor erlianensis* holotype. At least three foramina are located on the lateral surface of the lateral flange on both dentaries. In MPC-D 107/17, three relatively large foramina are also reported at a similar position near the lateral flange ([8]: Fig. 2). This supports the suggestion that MPC-D 107/17 is closely related to *Gigantoraptor* [8].

The anteroposterior length of the external mandibular fenestra is ~15.2cm and its maximum height is ~7.4cm, based on the left dentary. Its length is about one-third of the anteroposterior length of the mandible, which is proportionally shorter than that of *Anzu wyliei* ([6]: Fig. 2F) and *Caenagnathus collinsi* ([5]: Fig. 1). As in other caenagnathids, but unlike oviraptorids, no process of the surangular extends anteriorly to divide the fenestra.

The Meckelian groove is located near the ventral margin of the dentary. It is exposed in medial view and terminates on the inside of the dentary. It is different from more crownward caenagnathids where the groove terminates on the ventral surface of the dentary ([9]:character state 1 for character 187). No hour-glass shaped depression is present on the ventral surface of the symphysis in *Gigantoraptor*, unlike a number of crownward caenagnathids including *Leptorhynchos gaddisi* [10], *Leptorhynchos elegans* [10], *Chirostenotes pergracilis* [7] and *Caenagnathasia martinsoni* [5]. However, the possibility that the depression was affected by deformation cannot be ruled out.

**Supplementary Table S1. Mandibular measurements of *Gigantoraptor erlianensis* holotype LH V0011**

| **Measurements** | **Dimensions in cm** |
| --- | --- |
| **General** |  |
| Total mandibular length | 46.0 |
| Maximum mandibular height | 15.4 |
|  |  |
| **Dentary** |  |
| Dorsoventral depth at dentary-surangular contact | 13.5* (left) |
| Length from symphysis to external mandibular fenestra | 19.5 (left) |
| Anteroposterior length of symphysis | 7.6 |
| Height behind symphysis | 10.8 |
| Width behind symphysis | 10.0 |
|  |  |
| **External mandibular fenestra** |  |
| Maximum height of external mandibular fenestra | 7.4* (left) |
| Anteroposterior length of external mandibular fenestra | 15.2* (left) |
|  |  |
| **Articular-surangular-coronoid (ASC) complex** |  |
| Anteroposterior length of articular glenoid | 6.4 (left), 6.2 (right) |
| Mediolateral width of articular glenoid | 6.9 (left), 6.6 (right) |
| Mediolateral width of lateral facet of glenoid | 2.4 (left), 2.4 (right) |
| Mediolateral width of medial facet of glenoid | 4.5 (left), 4.2 (right) |
| Length between coronoid process and articular glenoid | 14.0 (left) |
| Length of retroarticular process | 1.8 (left) |
|  |  |
| **Angular** |  |
| Anteroposterior length | 21.4 (left) |

*estimated measurement

**Supplementary Table S2. Dentary measurement relative to total mandibular length of selected caenagnathid mandibles**

| **Caenagnathid specimen** | **Length from symphysis to external mandibular fenestra / total mandibular length** | **Height behind symphysis/ total mandibular length** | **Data source** |
| --- | --- | --- | --- |
| *Gigantoraptor erlianensis* (LH V0011) | 0.424 | 0.235 | LH V0011 |
| *Caenagnathus collinsi* (CMN 8776) | 0.462 | 0.0773 | [7]: Table 1  [5]: Table 1 |
| *Chirostenotes pergracilis* (TMP 2001.12.12) | 0.381 | 0.108 | [7]: Table 1 |

**Supplementary Table S3. Relative beak depth of oviraptorosaur mandibles** Measurements taken directly from *Gigantoraptor* or indirectly from publication text and figures.

| **Oviraptorosaur specimen** | **Estimated relative beak depth (X/Y)** | **Data source** |
| --- | --- | --- |
| *Microvenator celer* (AMNH 3041) | 1.32 | AMNH 3041 |
| *Gigantoraptor erlianensis* (LH V0011) | 1.42 | LH V0011 |
| *Anzu wyliei* (CM 78000) | <0.45* | [6]: Fig. 3 |
| *Caenagnathus collinsi* (CMN 8776) | 0.28 | [5]: Table 1 |
| *Caenagnathasia martinsoni* (CMGP 401/12457) | 0.74 | [5]: Table 1 |
| *Chirostenotes pergracilis* (TMP 2001.12.12) | 0.62 | [7]: Table 1 |
| *Leptorhynchos elegans* (TMP 1992.36.390) | 0.62 | [7]: Table 1 |
| *Leptorhynchos gaddisi* (TMM 45920-1) | ~0.78 | [10]: Fig. 8 |
| *Caenagnathasia sp.* (IVPP V 20377) | 0.66 | [15]: Table 1 |
| MPC-D 107/17 | ~1.36 | [8]: Fig. 2 |
| MPC-D 102/107 | ~1.0 | [16]: Fig. 2 |
| *Citipati osmolskae* (IGM 100/978) | 1.92 | [17]: Fig. 9 |
| *Khaan mckennai* (IGM 100/973) | 1.37 | [18]: Fig. 3 |
| *Nemegtomaia barsboldi* (GIN10012112) | 1.79 | [19]: Fig. 3 |
| *Yulong mini* (reconstruction based on HGM 41HIII-0107, HGM 41HIII-0108, HGM 41HIII-0109 & HGM 41HIII-0110) | 1.07 | [20]: Fig. 2 |
| *Incisivosaurus gauthieri* (IVPP V13326) | 0.67 | [21]: Fig. 1 |

*most anterior portion of the dentary is not preserved

**Supplementary Table S4. Relative position of coronoid process prominence of oviraptorosaur mandibles.** The mechanical advantage of the jaw-closing system related to the position of coronoid process prominence is defined as (effort distance / load distance), which is equivalent to (anteroposterior length between coronoid process prominence and articular glenoid / anteroposterior length between anterior tip of mandible and articular glenoid). Measurements were taken directly from *Gigantoraptor* and indirectly from text and figures in the literature.

| **Oviraptorosaur specimen** | **Relative position of coronoid process prominence** (anteroposterior length between coronoid process prominence and articular glenoid / total mandibular length) | **Mechanical advantage** (anteroposterior length between coronoid process prominence and articular glenoid / anteroposterior length between anterior tip of mandible and articular glenoid) | | **Data source** |
| --- | --- | --- | --- | --- |
| *Gigantoraptor erlianensis* (LH V0011) | 0.30 | | 0.34 | LH V0011 |
| *Caenagnathus collinsi* (CMN 8776) | 0.26 | | 0.30 | [5]: Fig. 1 |
| *Chirostenotes pergracilis* (TMP 2001.12.12) | 0.31 | | 0.35 | [7]: Fig. 3 |
| *Citipati osmolskae* (IGM 100/978) | 0.37 | | 0.47 | [17]: Fig. 9 |
| *Khaan mckennai* (IGM 100/973) | 0.51 | | 0.52 | [18]: Fig. 3 |
| *Nemegtomaia barsboldi* (GIN10012112) | 0.32 | | 0.37 | [19]: Fig. 3 |
| *Yulong mini* (reconstruction based on HGM 41HIII-0107, HGM 41HIII-0108, HGM 41HIII-0109 & HGM 41HIII-0110) | 0.29 | | 0.33 | [20]: Fig. 2 |
| *Incisivosaurus gauthieri* (IVPP V13326) | 0.33 | | 0.39 | [21]: Fig. 1 |

**Supplementary Table S5. Degree of symphysis deflection of oviraptorosaur mandibles** Measurements taken directly from *Gigantoraptor* and indirectly from text and figures in the literature.

| **Oviraptorosaur specimen** | **Degree of dentary symphysis deflection / °** | **Data source** |
| --- | --- | --- |
| *Gigantoraptor erlianensis* (LH V0011) | 7 | LH V0011 |
| *Anzu wyliei* (CM 78000) | 7 | [6]: Fig. 2 |
| *Caenagnathus collinsi* (CMN 8776) | ~0 | [5]: Fig. 1 |
| *Chirostenotes pergracilis* (TMP 2001.12.12) | ~0 | [7]: Fig. 3 |
| *Citipati osmolskae* (IGM 100/978) | 34 | [17]: Fig. 9 |
| *Khaan mckennai* (IGM 100/973) | 20 | [18]: Fig. 3 |
| *Nemegtomaia barsboldi* (GIN10012112) | 28 | [19]: Fig. 3 |
| *Yulong mini* (reconstruction based on HGM 41HIII-0107, HGM 41HIII-0108, HGM 41HIII-0109 & HGM 41HIII-0110) | 23 | [20]: Fig. 2 |
| *Incisivosaurus gauthieri* (IVPP V13326) | ~0 | [21]: Fig. 1 |

**Supplementary References**

1. Currie, P.J. and D.A. Eberth, *Palaeontology, sedimentology and palaeoecology of the Iren Dabasu Formation (Upper Cretaceous), Inner Mongolia, People's Republic of China.* Cretaceous Research, 1993. **14**(2): p. 127-144.

2. Xing, H., et al. *A review on the study of the stratigraphy, sedimentology, and paleontology of the Iren Dabasu Formation, Inner Mongolia*. 2012. Proceedings of the Thirteenth Annual Meeting of the Chinese Society of Vertebrate Paleontology. Beijing: China Ocean Press.

3. Van Itterbeeck, J., et al., *Stratigraphy and palaeoenvironment of the dinosaur-bearing Upper Cretaceous Iren Dabasu Formation, Inner Mongolia, People's Republic of China.* Cretaceous Research, 2005. **26**(4): p. 699-725.

4. Bonnetti, C., et al., *Sedimentology, stratigraphy and palynological occurrences of the late Cretaceous Erlian Formation, Erlian Basin, Inner Mongolia, People's Republic of China.* Cretaceous Research, 2014. **48**: p. 177-192.

5. Currie, P.J., S.J. Godfrey, and L. Nessov, *New caenagnathid (Dinosauria: Theropoda) specimens from the Upper Cretaceous of north America and Asia.* Canadian Journal of Earth Sciences, 1993. **30**(10): p. 2255-2272.

6. Lamanna, M.C., et al., *A new large-bodied oviraptorosaurian theropod dinosaur from the latest Cretaceous of western North America.* PloS one, 2014. **9**(3): p. e92022.

7. Funston, G.F. and P.J. Currie, *A previously undescribed caenagnathid mandible from the late Campanian of Alberta, and insights into the diet of Chirostenotes pergracilis (Dinosauria: Oviraptorosauria).* Canadian Journal of Earth Sciences, 2014. **51**(2): p. 156-165.

8. Tsuihiji, T., et al., *A gigantic caenagnathid oviraptorosaurian (Dinosauria: Theropoda) from the Upper Cretaceous of the Gobi Desert, Mongolia.* Cretaceous Research, 2015. **56**: p. 60-65.

9. Funston, G.F. and P.J. Currie, *A new caenagnathid (Dinosauria: Oviraptorosauria) from the Horseshoe Canyon Formation of Alberta, Canada, and a reevaluation of the relationships of Caenagnathidae.* Journal of Vertebrate Paleontology, 2016: p. e1160910.

10. Longrich, N.R., et al., *Caenagnathidae from the Upper Campanian Aguja Formation of West Texas, and a Revision of the Caenagnathinae.* Bulletin of the Peabody Museum of Natural History, 2013. **54**(1): p. 23-49.

11. Rozzi, R. and J.E. Jiménez, *Magellanic Sub-Antarctic Ornithology: First Decade of Long-term Bird Studies at the Omora Ethnobotanical Park, Cape Horn Biosphere Reserve, Chile*. 2014: University of North Texas Press.

12. Grant, P.R. and B.R. Grant, *40 years of evolution: Darwin's finches on Daphne Major island*. 2014: Princeton University Press.

13. Badyaev, A.V., *The beak of the other finch: coevolution of genetic covariance structure and developmental modularity during adaptive evolution.* Philosophical Transactions of the Royal Society of London B: Biological Sciences, 2010. **365**(1543): p. 1111-1126.

14. Eck, S., et al., *Measuring birds–Vögel Vermessen*. 2011, Wihelmshaven: Deutsche Ornithologen-Gesellschaft.

15. Yao, X., et al., *Caenagnathasia sp (Theropoda: Oviraptorosauria) from the Iren Dabasu Formation (Upper Cretaceous: Campanian) of Erenhot, Nei Mongol, China.* Vertebrata Palasiatica, 2015. **53**(4): p. 291-298.

16. Tsuihiji, T., et al., *Dentaries of a caenagnathid (Dinosauria: Theropoda) from the Nemegt Formation of the Gobi Desert in Mongolia.* Cretaceous Research, 2016. **63**: p. 148-153.

17. Clark, J.M., M.A. Norell, and T. Rowe, *Cranial anatomy of Citipati osmolskae (Theropoda, Oviraptorosauria), and a reinterpretation of the holotype of Oviraptor philoceratops.* American Museum Novitates, 2002(3364): p. 1-24.

18. Balanoff, A.M. and M.A. Norell, *Osteology of Khaan mckennai (Oviraptorosauria: Theropoda).* Bulletin of the American Museum of Natural History, 2012(372): p. 1-76.

19. Lü, J., et al., *New oviraptorid dinosaur (Dinosauria: Oviraptorosauria) from the Nemegt Formation of southwestern Mongolia.* Bulletin of the National Science Museum Series C (Geology & Paleontology), 2004. **30**: p. 95-130.

20. Lü, J., et al., *Chicken-sized oviraptorid dinosaurs from central China and their ontogenetic implications.* Naturwissenschaften, 2013. **100**(2): p. 165-175.

21. Xu, X., et al., *An unusual oviraptorosaurian dinosaur from China.* Nature, 2002. **419**.
